# Investigation on the Microstructure and Compressive Properties of W-15Mo Alloy Fabricated via Laser Powder Bed Fusion

**DOI:** 10.3390/ma19163376

**Published:** 2026-08-08

**Authors:** Yuting Song, Yujie Ao, Qiao Deng, Linwei Zhang, Jilin Liu, Zichun Wu, Jiancheng Tang, Nan Ye

**Affiliations:** 1 School of Physics and Materials Science, Nanchang University, Nanchang 330031, China; chenyu_song@ncu.edu.cn (Y.S.); 15083639823@163.com (Y.A.); 13873523447@163.com (Q.D.); 18827865381@163.com (J.L.);; 2 Institute of Materials and Intelligent Manufacturing, Jiangxi Academy of Sciences, Nanchang 330095, China; 3 International Institute for Materials Innovation, Nanchang University, Nanchang 330031, China; tangjiancheng@ncu.edu.cn

**Keywords:** W-Mo alloy, laser powder bed fusion, annealing, compressive performance

## Abstract

Solid solution with molybdenum is an effective strategy for improving the overall performance of tungsten alloys, thereby enhancing their application potential in aerospace and other fields. In this study, highly dense W-Mo alloy specimens were successfully fabricated using laser powder bed fusion, followed by stress-relief annealing. The results show that the complete solid solution of molybdenum yields a fine columnar grain structure with weak texture. After stress-relief annealing, the residual stress decreases, the grain size increases slightly, and the fraction of the <111>//building direction (BD) texture rises. Ultimately, W-Mo alloy specimens with excellent compressive properties are obtained, with compressive yield strength, ultimate compressive strength, and compressive strain reaching 879.5 MPa, 1257.3 MPa, and 16.6%, respectively. This work provides new insights into the fabrication of refractory alloys by laser powder bed fusion.

## 1. Introduction

Tungsten and its alloys are widely used in aerospace, electronic and chemical engineering, and nuclear industries, owing to their high melting point, high elastic modulus, and excellent high-temperature strength [1,2,3]; typical applications include turbine components, spallation neutron source targets, and fusion reactor target materials. However, their practical applications are severely constrained by a high ductile-to-brittle transition temperature (DBTT), recrystallization embrittlement, and poor oxidation resistance [4]. An effective strategy to address these issues is the formation of an infinite solid solution by adding molybdenum, which shares the same BCC structure as tungsten [5]. Molybdenum also possesses a relatively high melting point and outstanding high-temperature performance [6]. Previous studies have demonstrated that W-Mo solid solutions can effectively improve hardness, strength, toughness, and other high-temperature mechanical properties through solid-solution strengthening [2,7,8]. Nevertheless, conventional manufacturing processes for W-Mo alloys, such as powder metallurgy and vacuum melting, are increasingly inadequate for meeting the growing demand for complex-shaped structural components, as these routes are generally limited to simple geometries and require extensive post-machining [2]. Therefore, innovative approaches are urgently needed to enable the efficient fabrication of intricate W-Mo alloy parts.

Laser powder bed fusion (LPBF) is an advanced additive manufacturing technique that builds complex components by selectively melting metal powders layer by layer based on a digital model [9,10]. Hence, employing this technique to fabricate complex and irregular W-Mo alloy components holds promise for overcoming the limitations of conventional manufacturing routes, enabling the production of high-performance and intricate W-Mo alloy parts. To date, significant progress has been made in the LPBF processing of both tungsten-based and molybdenum-based alloys. Sidambe et al. [11] fabricated pure tungsten bulk samples via LPBF and investigated the influence of laser energy density on densification, they obtained samples with a relative density of 97% under optimized conditions. Ma et al. [12] studied the effects of processing parameters on the microstructure and mechanical properties of LPBF-produced pure molybdenum, achieving a density exceeding 95% and a tensile strength of 117.18 MPa. However, research on the microstructure and properties of W-Mo alloys fabricated by LPBF remains relatively scarce.

In this work, a high-density W-15Mo alloy specimen was fabricated by LPBF, followed by stress-relief annealing. The microstructural characteristics, grain morphology, crystallographic texture, and mechanical properties of both the as-printed and heat-treated specimens were systematically investigated using X-Ray Diffraction (XRD), optical microscope (OM), scanning electron microscope (SEM), electron backscatter diffraction (EBSD), and compression tests. This study provides a methodological reference for the LPBF fabrication and stress-relief treatment of refractory alloys.

## 2. Experimental

### 2.1. Raw Powers

A W-Mo alloy powder with a particle size range of 15 μm–53 μm, synthesized via spray drying combined with RF plasma spheroidization, was employed as the starting material. Prior to printing, the powder was dried in a DZF-6090 vacuum oven (Shanghai Yiheng Technology Co., Ltd., Shanghai, China) at 120 °C for 5 h to eliminate residual moisture.

### 2.2. LPBF Process and Robust Process Design

The LPBF experiments were conducted on a BLT-S210 machine (Xi’An Bright Laser Technologies Co., Ltd., Xi’An, China) equipped with a YLR-500-SM fiber laser (wavelength 1070 nm, fixed beam diameter 100 μm), with a 316 stainless steel plate serving as the substrate. The initial oxygen content in the chamber was maintained below 100 ppm, and the substrate was preheated to 250 °C to mitigate residual stress accumulation. A layer rotation angle of 67° was adopted to minimize thermal stress buildup during the build process [13]. To further investigate the contribution of process parameters and identify the process parameter window for achieving high relative density specimens, the Taguchi method and response surface methodology were adopted. Robust process design was performed over the following ranges: laser power 200 W-275 W, scanning speed 200 mm/s–800 mm/s, hatch spacing 0.10 mm, and layer thickness 0.03 mm. An L_16_ orthogonal array was generated using Minitab^®^ version 17. The experimental results were converted into signal-to-noise (S/N) ratios to evaluate both the mean performance and the dispersion of the measurements. By maximizing the S/N ratio, process parameters that enhance performance while reducing quality variation can be identified. Since microstructural defects generally lead to performance degradation, relative density was selected as the S/N response parameter. The S/N ratio is commonly classified into three categories: larger-the-better, smaller-the-better, and nominal-the-better. As the objective of this study was to achieve the maximum relative density, the larger-the-better criterion was adopted [14]:

(1) $$\frac{S}{N}=-10\log(\frac{1}{n}\sum_{i=1}^{n}\frac{1}{y_{i}^{2}})$$

where *n* is the number of experiments and *y_i_* is the relative density of the *i*-th experiment. Subsequently, analysis of variance (ANOVA) was performed to determine the significance of each process parameter. After obtaining the specimen with the highest relative density, the samples were subjected to stress-relief annealing in a BTF-1700C tube furnace (Anhui BEQ Equipment Technology Co., Ltd., Hefei, China) under an argon atmosphere at 1200 °C for 2 h. Cuboidal specimens of 10 mm × 10 mm × 10 mm were prepared for microstructural characterization, while cylindrical specimens of 8 mm diameter and 12 mm height were fabricated for compression testing.

### 2.3. Microstructure Characterization and Mechanical Property Test

Cuboidal specimens of 10 mm × 10 mm × 10 mm were prepared for microstructural characterization. Density measurements were performed using the Archimedes method in accordance with ASTM B311 standard [15]. XRD analysis was conducted on a Rigaku D/max-A diffractometer equipped with Cu Kα radiation (λ = 1.5406 Å), operated at 40 kV and 40 mA, and the diffraction patterns were recorded over a 2θ range of 20° to 90° with a step size of 0.05°. Microstructural features were characterized using a Leica DMI8A optical microscope (OM), a Zeiss Gemini 300 scanning electron microscope (SEM), and an electron backscatter diffraction (EBSD) system. Vickers hardness was measured using an HV-1000 tester under a load of 0.2 kgf and a dwell time of 15 s. For each sample, five indentations were randomly made at different locations, and the average value was calculated. Cylindrical specimens of Ø8 mm × 12 mm were prepared for compression testing. Room-temperature compression tests were conducted on an Instron 3369 universal testing machine at a crosshead speed of 1 mm/min, with the loading direction parallel to the building direction, to obtain the compressive properties.

## 3. Results

### 3.1. Microstructure of W-Mo Alloy Powder

**Figure 1:**
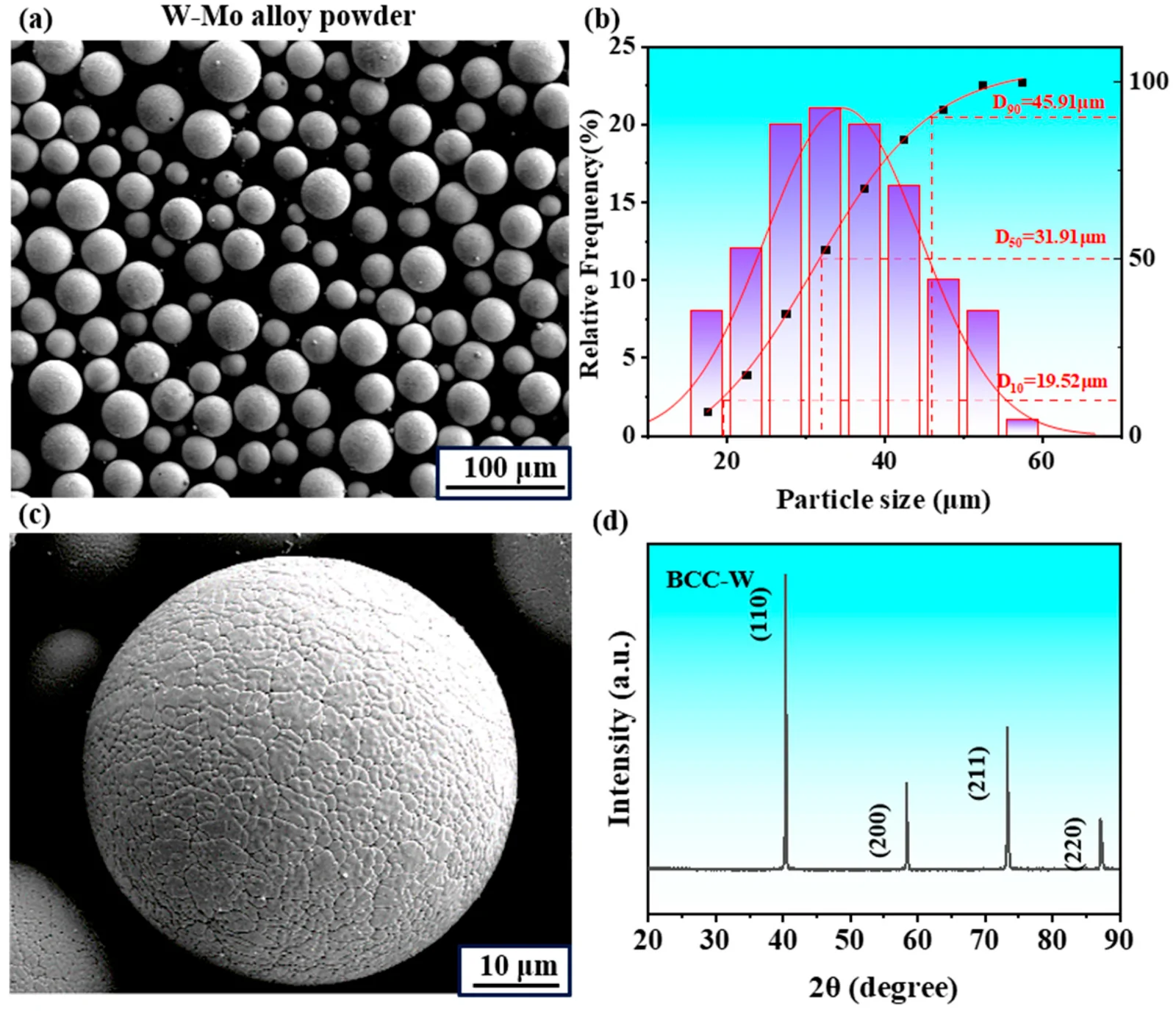
Characterization of the W-Mo alloy powder: (**a**) high-magnification SEM image, (**b**) particle size distribution, (**c**) low-magnification SEM image and (**d**) XRD pattern.

The powder morphology and particle size distribution (the black square points represent the center of the interval), shown in Figure 1a–c, reveal a high degree of sphericity, an absence of hollow particles, and a narrow size distribution. The XRD pattern (Figure 1d) of the powder exhibits a single BCC phase, indicating the complete dissolution of Mo into the W matrix following the combined spray drying and RF plasma spheroidization process.

### 3.2. Robust Process Design of As-Built W-Mo Alloy

In the LPBF process, a high relative density in the microstructure of the samples is generally desired, as a higher level of microstructural defects can readily act as crack nucleation sites, leading to premature failure of the sample. Table 1 presents the specific combinations of the L_16_ orthogonal array, the corresponding energy density, and the measured relative density. Here, the laser energy density is calculated according to the following formula:

(2) $$E_{v}=\frac{P}{vht}$$

where *P* is the laser power (W), *v* is the scanning speed (mm/s), *h* represents the scanning interval (mm), *t* is the powder-bed layer thickness.

Through robust process design, a sample with a relative density of 99.46% was obtained at a laser power of 250 W, a scanning speed of 400 mm/s, a fixed layer thickness of 0.03 mm, a hatch spacing of 0.10 mm, and a laser energy density of 208.33 J/mm^3^.

**Figure 2:**
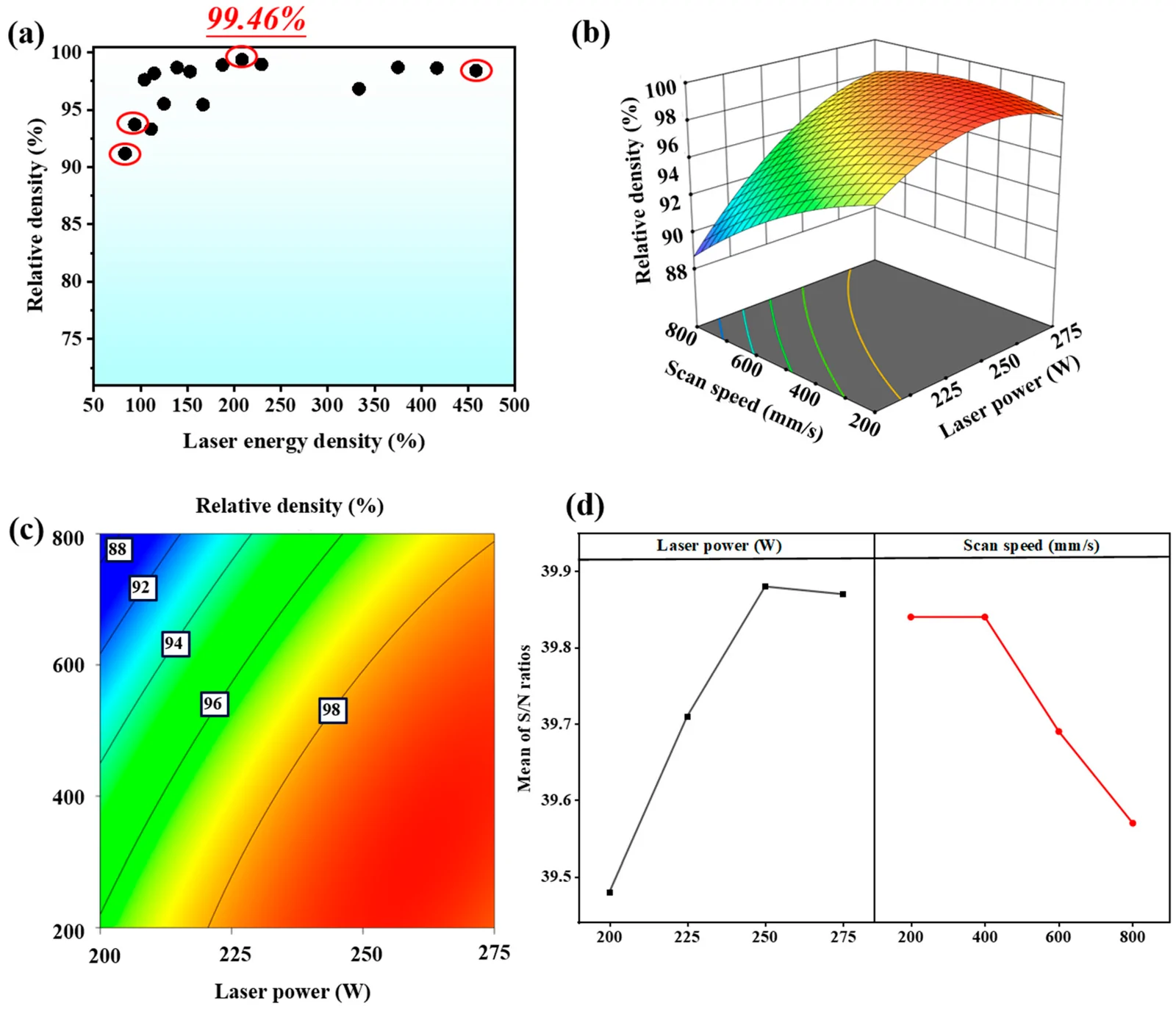
Robust process design of the W-Mo alloy: (**a**) relationship between relative density and energy density, (**b**) response surface graph showing the effect of the laser power and scan speed on relative density, (**c**) contour plot of the effect of the laser power and scan speed on relative density, (**d**) main effect plots of S/N ratio-relative density.

Both the laser energy density and the individual process parameters were found to exert a significant influence on the relative density of the samples. The variation in relative density with laser energy density is shown in Figure 2a. As the laser energy density increases, the relative density first rises and then gradually decreases. At energy densities below 100 J/mm^3^, the relative density remains low, below 95%. When the energy density exceeds 100 J/mm^3^, a considerable improvement in relative density is observed, although a few isolated points still exhibit lower values. At energy densities above 250 J/mm^3^, the relative density consistently falls below 99%, indicating a reduced level of densification. The response surface and contour plots of relative density as a function of laser power and scanning speed are presented in Figure 2b,c, respectively. Relative density increases with increasing laser power, whereas it decreases with increasing scanning speed. Combining these trends with the S/N ratio results (Table 2) and Figure 2d, it is evident that laser power exerts a greater influence on relative density than scanning speed. Moreover, the highest S/N ratios for relative density were obtained at a laser power of 250 W and a scanning speed of 400 mm/s. The contributions of the two process parameters to the relative density, determined by ANOVA, are listed in Table 3. Laser power accounts for 56.13% of the contribution, while scanning speed accounts for 28.03%, confirming the more dominant role of laser power. This also accounts for the observed variation in relative density under nominally identical laser energy density conditions.

Subsequently, the experimental results were imported into the Design-Expert^®^ version 13 software for regression analysis. The regression equation between the relative density and the process parameters (laser power and scanning speed) can be expressed as [14]:

(3) $$Relative\;density=a_{0}+a_{1}(A)+a_{2}(B)+a_{3}(AB)$$

**Figure 3:**
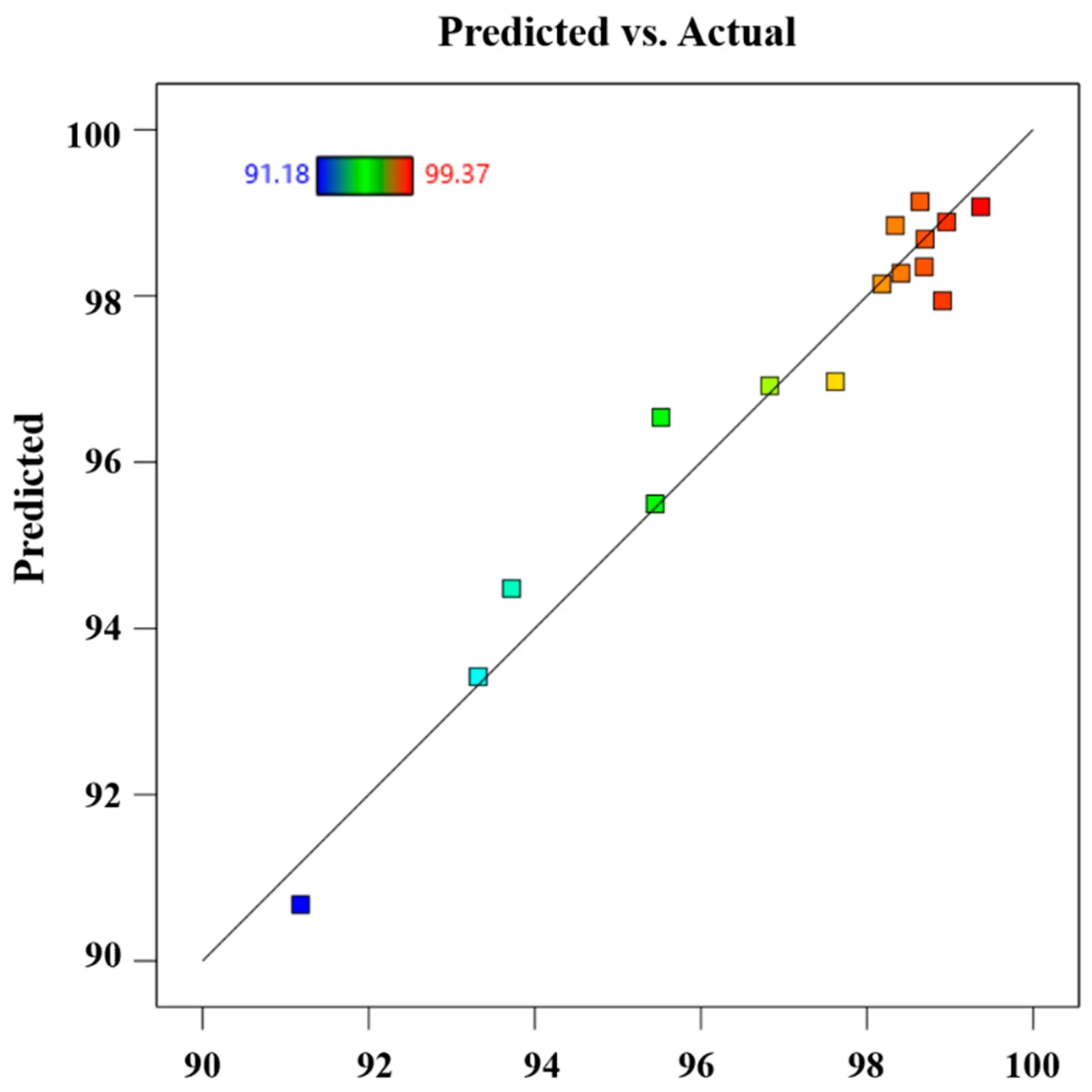
Correlation graph of the experimental values and predicted values of relative density.

The corresponding coefficient values *a*_0_ to *a*_3_ are presented in Table 4, *A* and *B* represent the laser power and scanning speed, respectively. By substituting these values, the predicted relative density values are obtained. The predicted relative densities for each parameter combination are shown in Table 1 and Figure 3. Upon comparison, it is found that the predicted relative density values exhibit a high correlation with the experimental values.

This non-monotonic relationship between laser energy density and densification is not unique to the W-Mo alloy system but rather represents a general principle governing the densification behaviour of LPBF-processed alloys. For instance, in Ti6Al4V, the relative density progressively increases with laser energy density from 33 J/mm^3^ to approximately 90 J/mm^3^, reaching a maximum of 99.43%, while further increasing laser energy density beyond 100 J/mm^3^ results in a slight decrease in density, attributable to the transition from lack of fusion at low energy inputs to keyhole porosity at excessive energy inputs [16]. For a nickel-based superalloy, the porosity can be maintained below 0.68% when laser energy density falls within the range of 47 J/mm^3^–78 J/mm^3^, and near-full density (porosity~0.04%) is achieved under optimized conditions; however, excessively high laser power and scanning speed lead to deformation and print failure [17]. For the 2024 Al alloy, either insufficient laser energy density (≤40 J/mm^3^) or excessive laser energy density (≥120 J/mm^3^) causes a marked reduction in density, with porosity increasing sharply to 8%, corresponding to irregular pores or large cavities, respectively [18]. These findings collectively demonstrate that the correlation between laser energy density and densification is a fundamental processing criterion common to various LPBF-manufactured alloys.

**Figure 4:**
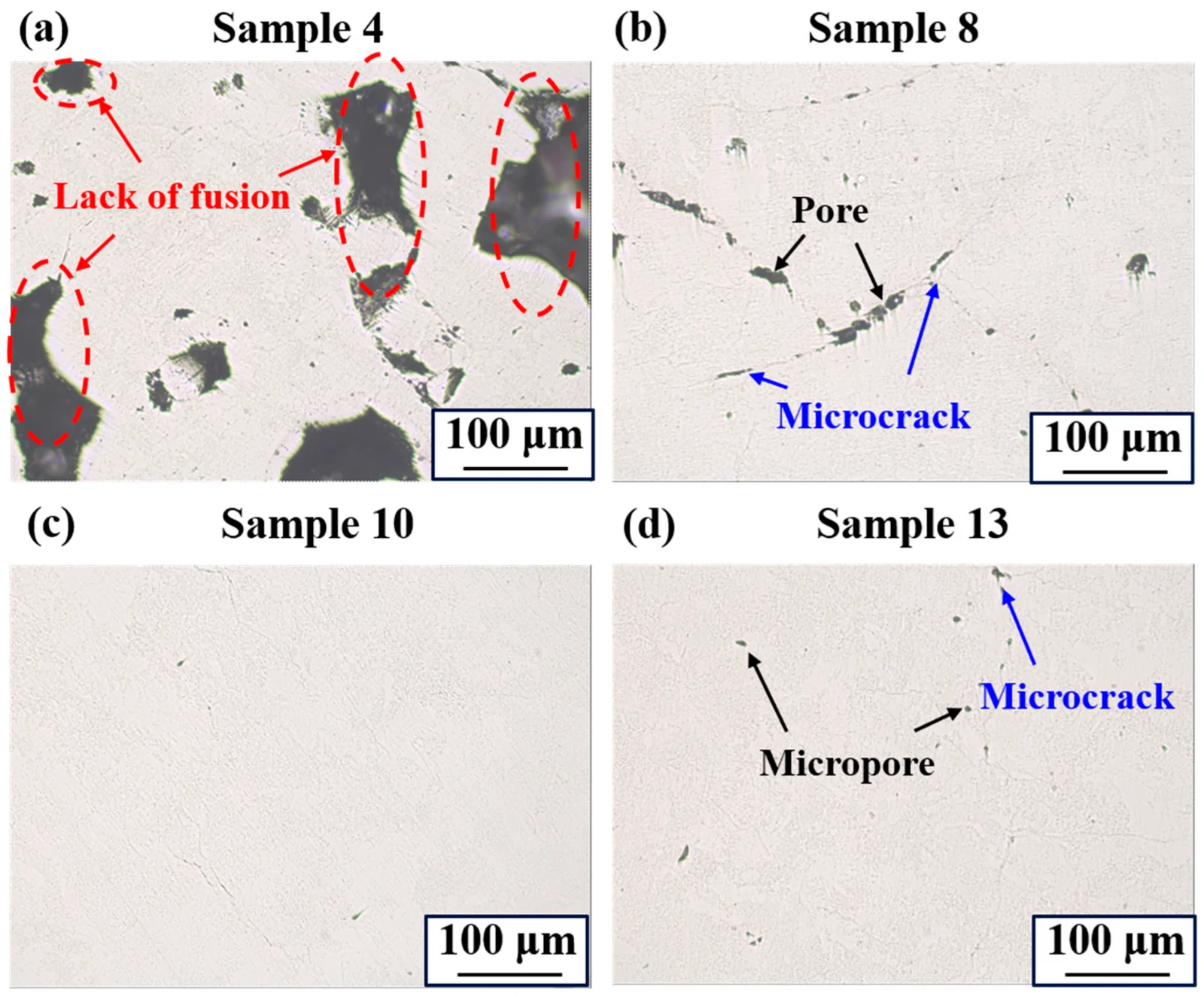
Microstructural evolution of the W-Mo alloy under different laser energy densities: (**a**) 91.18 J/mm^3^, (**b**) 125 J/mm^3^, (**c**) 208.33 J/mm^3^, (**d**) 458.33 J/mm^3^.

Figure 4 shows the OM images of the W-Mo alloy under different laser energy densities. At a laser energy density of 91.18 J/mm^3^, a large number of large-sized and irregularly shaped lack-of-fusion defects are observed. As the laser energy density increases to 125 J/mm^3^, the size and number of pores gradually decrease, and the pore morphology becomes more regular, although some micro-cracks and pores still exist. When the laser energy density is further increased to 208.33 J/mm^3^, the porosity and pore size are further reduced, exhibiting a highly dense microstructure. However, when the laser energy density is increased to 458.33 J/mm^3^, the relative density decreases, accompanied by a small number of micropores and microcrack defects.

In the LPBF process, lack of fusion, gas pores, and microcracks are the main types of defects [19,20,21]. When the laser energy density is too low, there is insufficient laser energy input, the melt pool spreading time is too short, and some powder remains unmelted. An appropriately increased laser energy density improves the melting efficiency of the melt pool, increases the melt pool spreading time, and promotes the overlapping and fusion of scan tracks, thereby increasing the relative density of the sample. However, excessively high laser energy density can complicate the melt pool flow, causing turbulence, which intensifies thermal shock, leads to melt pool spattering and more severe residual stress accumulation, resulting in defects such as micropores and cracks, thus impairing the mechanical properties of the sample [22,23].

### 3.3. Microstructures of As-Built W-Mo Alloys

**Figure 5:**
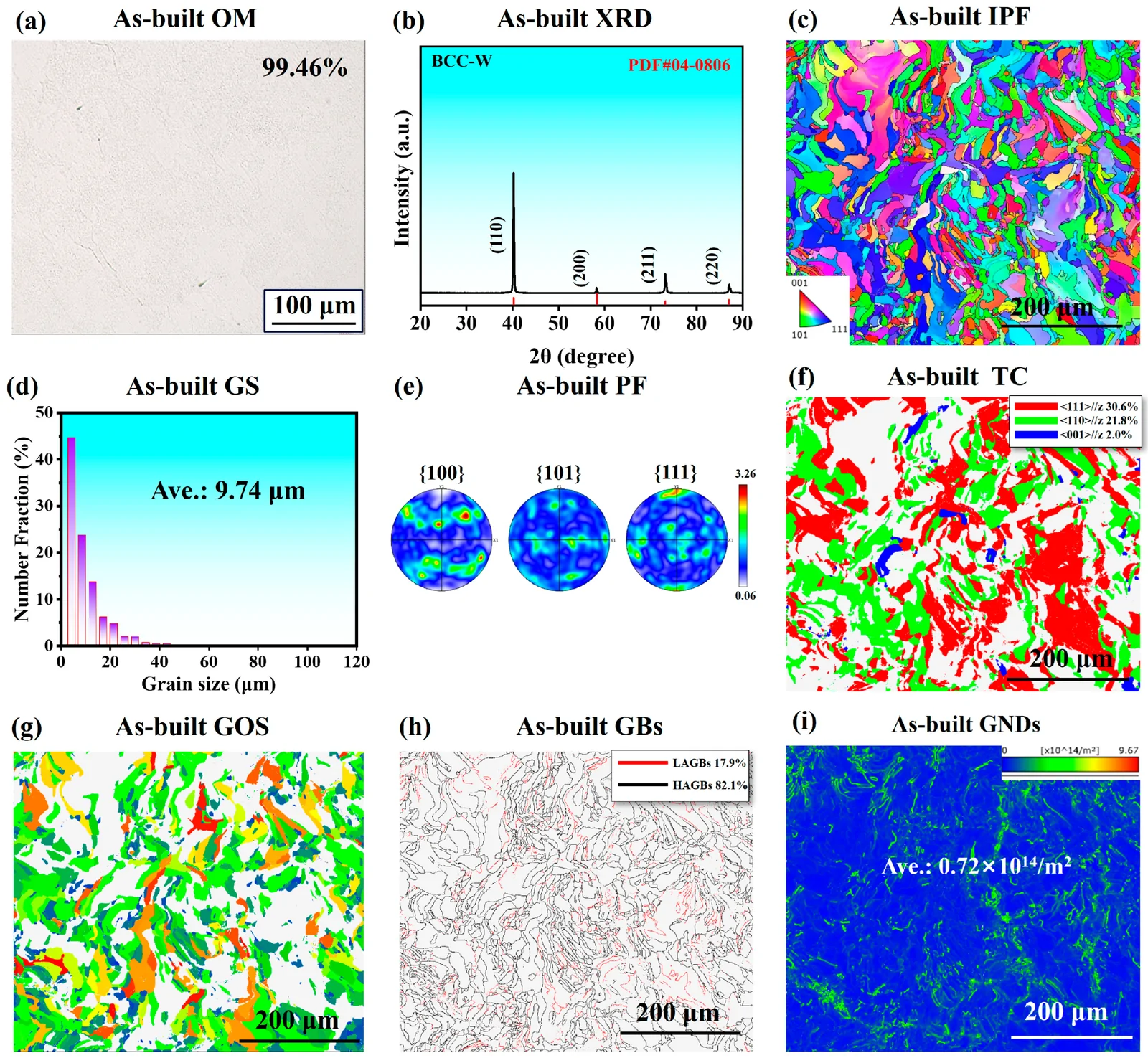
Microstructures of the as-built W-Mo alloys: (**a**) optical micrographs, (**b**) XRD, (**c**) IPF, (**d**) grain size distribution, (**e**) PF, (**f**) texture component parallel to the BD, (**g**) recrystallized grain maps defined by grain orientation spread (GOS) < 2°, (**h**) GBs, (**i**) GNDs.

The microstructures, XRD pattern, and EBSD results of the as-built W-Mo alloy are shown in Figure 5. As presented in Figure 5a, the alloy exhibited a high relative density of 99.3%, and no significant pores or microcracks were observed. The XRD pattern in Figure 5b displays only reflections corresponding to the BCC-W phase, confirming the complete solid solution of Mo. The characteristic peaks were recorded at 40.264° for (110), 58.274° for (200), 73.195° for (211), and 87.021° for (220). The inverse pole figures (red, green, and blue representing <001>, <101>, and <111>, respectively) together with the grain-size distribution maps Figure 5c,d indicate the absence of a pronounced crystallographic texture. The grain morphology consisted predominantly of narrow, elongated, curved columnar grains within the melt pools and fine equiaxed grains at the melt-pool boundaries, with an average grain size of 9.74 μm.

During additive manufacturing, cubic metals typically grow preferentially along the <100> crystal direction, which is parallel to the direction of maximum heat flux, thus tending to form coarse epitaxial columnar grains that penetrate multiple melt pools [24,25]. In contrast, the introduction of molybdenum atoms with unlimited solid solubility into tungsten creates a significant solute drag effect that continuously impedes grain boundary migration during solidification, thereby further inhibiting grain coarsening [26]. The formation of curved columnar grains is closely related to the thermodynamics and kinetics of the melt pool [27,28]. Owing to the temperature gradient difference between the horizontal direction and the building direction (BD), and because the heat flow direction is always perpendicular to the solid-liquid interface, the grain growth direction is opposite to the heat flow direction during solidification. This leads to anisotropic grain growth at the center and boundary of the melt pool. As the melt pool solidifies, the grain growth direction bends from the boundary toward the center, giving rise to these curved grains. Furthermore, the angle between the laser scanning direction and the melt pool center differs from that at the boundary, resulting in different grain growth velocities. Typically, equiaxed growth occurs along the centerline of the melt pool, while coarse columnar grains are readily formed near the melt pool boundary [29,30].

Figure 5e,f present the {100}, {101}, and {111} pole figures of the as-built W-Mo alloys, along with the corresponding fractions of the <001>, <101>, and <111> texture components parallel to the BD (represented by blue, green, and red rectangles, respectively). The as-built alloy exhibits preferred orientations of {100} and {111}, with a multiple of uniform distribution (MUD) value of 3.29, indicating a relatively low texture level. Regarding the texture components parallel to BD, the fractions of <001>, <110>, and <111> are 2.0%, 21.8%, and 30.6% in the as-built state. Figure 5g,h show the grain boundary maps (low-angle grain boundaries (LAGBs): 2–15°, high-angle grain boundaries (HAGBs): >15°) and the recrystallized grain maps defined by grain orientation spread (GOS) < 2° of the as-built W-Mo alloys. The fractions of LAGBs and HAGBs are 17.9% and 82.1%, respectively. The recrystallized grains revealed that the recrystallized volume fraction is 57.4% in the as-built state. The relatively high recrystallized volume fraction in the as-built condition originated mainly from the repeated thermal cycling inherent to the laser additive manufacturing process, which induced dynamic recrystallization in certain regions [31]. Figure 5i shows the geometrically necessary dislocation (GND) density of the as-built W-Mo alloys. The alloy exhibits a GND density of 0.72 × 10^14^ m^−2^, which is predominantly distributed at LAGBs. This is attributed to the ability of LAGBs to impede dislocations and arrange them in an orderly manner. Meanwhile, dislocations in the alloy are concentrated in localized regions.

### 3.4. Microstructures of As-Annealed W-Mo Alloys

**Figure 6:**
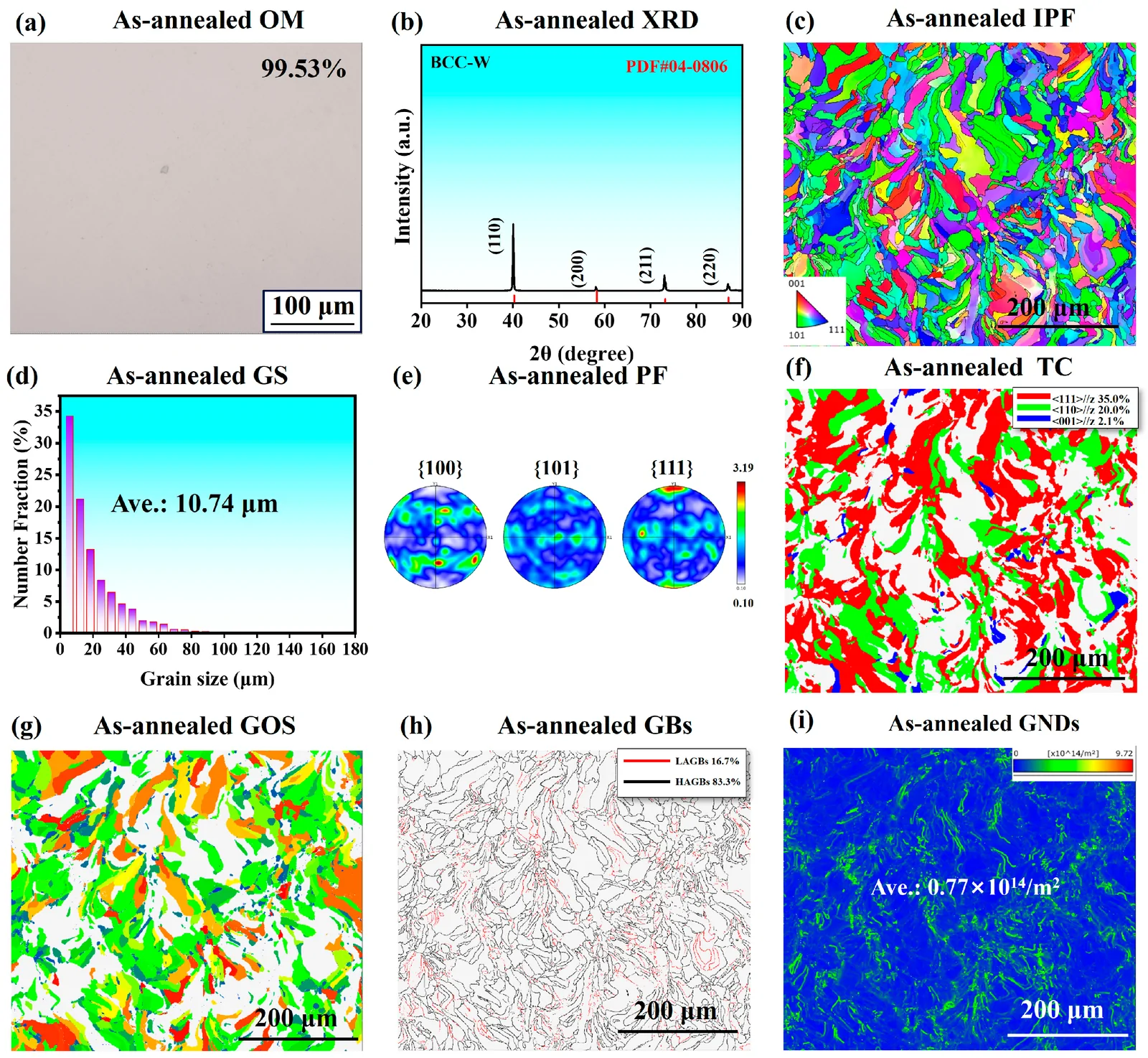
Microstructures of the as-annealed W-Mo alloys: (**a**) optical micrographs, (**b**) XRD, (**c**) IPF, (**d**) grain size distribution, (**e**) PF, (**f**) texture component parallel to the BD, (**g**) recrystallized grain maps defined by grain orientation spread (GOS) < 2°, (**h**) GBs, (**i**) GNDs.

The microstructures, XRD pattern, and EBSD results of the as-annealed W-Mo alloy are presented in Figure 6. As shown in Figure 6a, the annealed microstructure exhibited a slight reduction in defects compared with the as-built state, and the relative density reached 99.5%. The XRD pattern in Figure 6b revealed that no new phases were formed, although the diffraction peaks shifted to lower angles. According to Bragg’s law, this shift corresponds to an increased interplanar spacing, suggesting that annealing facilitated the release of residual stresses and the recovery of the equilibrium lattice parameter [32]. Moreover, the intensity of the (110) peak decreased considerably, indicating that the annealing treatment effectively weakened the crystallographic texture.

The inverse pole figures and grain size distribution maps (Figure 6c,d) showed that the annealed specimen also comprised columnar grains within the melt pools and equiaxed grains at the boundaries, with an average grain size of 10.74 μm. This marginal change indicates that annealing exerted only a limited effect on grain growth, reflecting the excellent thermal stability of the fabricated W-Mo alloy. The pole figures and texture components parallel to BD for the annealed alloy are shown in Figure 6e,f. The main preferred orientations remained {100} and {111}, with fractions of <001>//BD, <110>//BD, and <111>//BD of 2.1%, 20.0%, and 35.0%, respectively. These results indicate that annealing promoted the development of the <111>//BD texture, which is primarily attributable to the higher stored energy of the <111> texture in BCC metals, leading to a faster grain growth rate during annealing [33].

Furthermore, the fractions of LAGBs and high-angle grain boundaries HAGBs in the annealed alloy, shown in Figure 6h, were 16.7% and 83.3%, respectively. This indicates that the annealing temperature remained below the recrystallization temperature, resulting in only minor microstructural changes, with recovery being the dominant mechanism, this also corresponds to the recrystallized volume fraction of 55.7% shown in Figure 6g. During recovery, dislocations rearranged via glide and climb, and some LAGBs either disappeared or transformed into HAGBs through the rotation and coalescence of adjacent subgrains, leading to a slight increase in the HAGB fraction [34]. Further identification of recrystallized grains revealed that the recrystallized volume fraction in the annealed state was 55.7%, showing no increase compared with the as-built state, confirming that no significant recrystallization nucleation or growth was initiated at this annealing temperature. This observation suggests that the thermal activation was insufficient to drive the migration of HAGBs. The dominant mechanism at this annealing temperature was thus the glide, climb, and rearrangement of dislocations during recovery, primarily serving to relieve the residual stresses in the printed part [34]. After annealing, the W-Mo alloy exhibited a GND density (Figure 6i) of 0.77 × 10^14^ m^−2^, which is associated with the higher fraction of the <111>//BD component possessing relatively high stored energy. Furthermore, the dislocation distribution was more uniform than that observed in the as-built state.

### 3.5. Mechanical Properties of As-Printed and Annealed W-Mo Alloys

**Figure 7:**
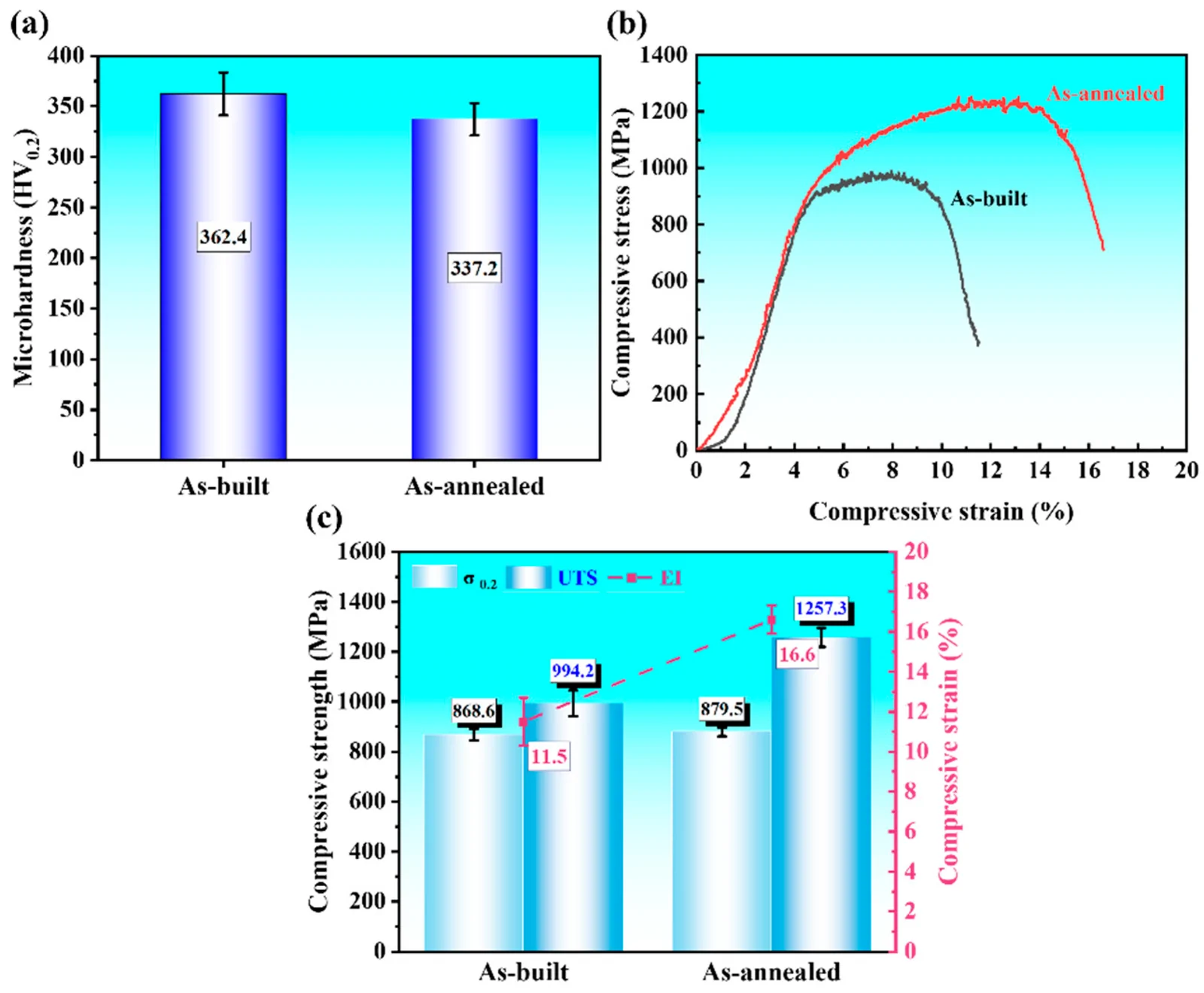
Mechanical properties of the as-built and annealed W-Mo alloys: (**a**) microhardness, (**b**) compressive stress-strain curves, and (**c**) comparison of compressive properties.

Figure 7 displays the microhardness and room-temperature compression test results of the as-built and annealed W-Mo alloys. As shown in Figure 7a, the Vickers hardness of the as-built alloy is 362.4 HV_0.2_, while that of the annealed alloy decreases to 337.2 HV_0.2_. According to the Hall-Petch equation, there is a linear relationship between the Vickers hardness and the inverse square root of the grain size [35]:

(4) $$HV=HV_{0}+K_{h}D^{-\frac{1}{2}}$$

where $K_{h}$ and $HV_{0}$ are material constants, HV is the Vickers hardness, and *D* is the grain size [34]. Based on the previous EBSD grain size analysis, the grain size increases after annealing, leading to a decrease in microhardness. In addition, the release of residual stress also contributes to the decrease in hardness [36].

The room-temperature compression test results indicate that both the ultimate compressive strength and total compressive strain of the annealed sample are significantly improved compared with those of the as-built sample. As shown in Figure 7b,c, the compressive yield strength, ultimate compressive strength, and compressive strain of the as-built W-Mo alloy are 868.6 MPa, 994.2 MPa, and 11.5%, respectively, while after annealing they increase to 879.5 MPa, 1257.3 MPa, and 16.6%, respectively.

**Figure 8:**
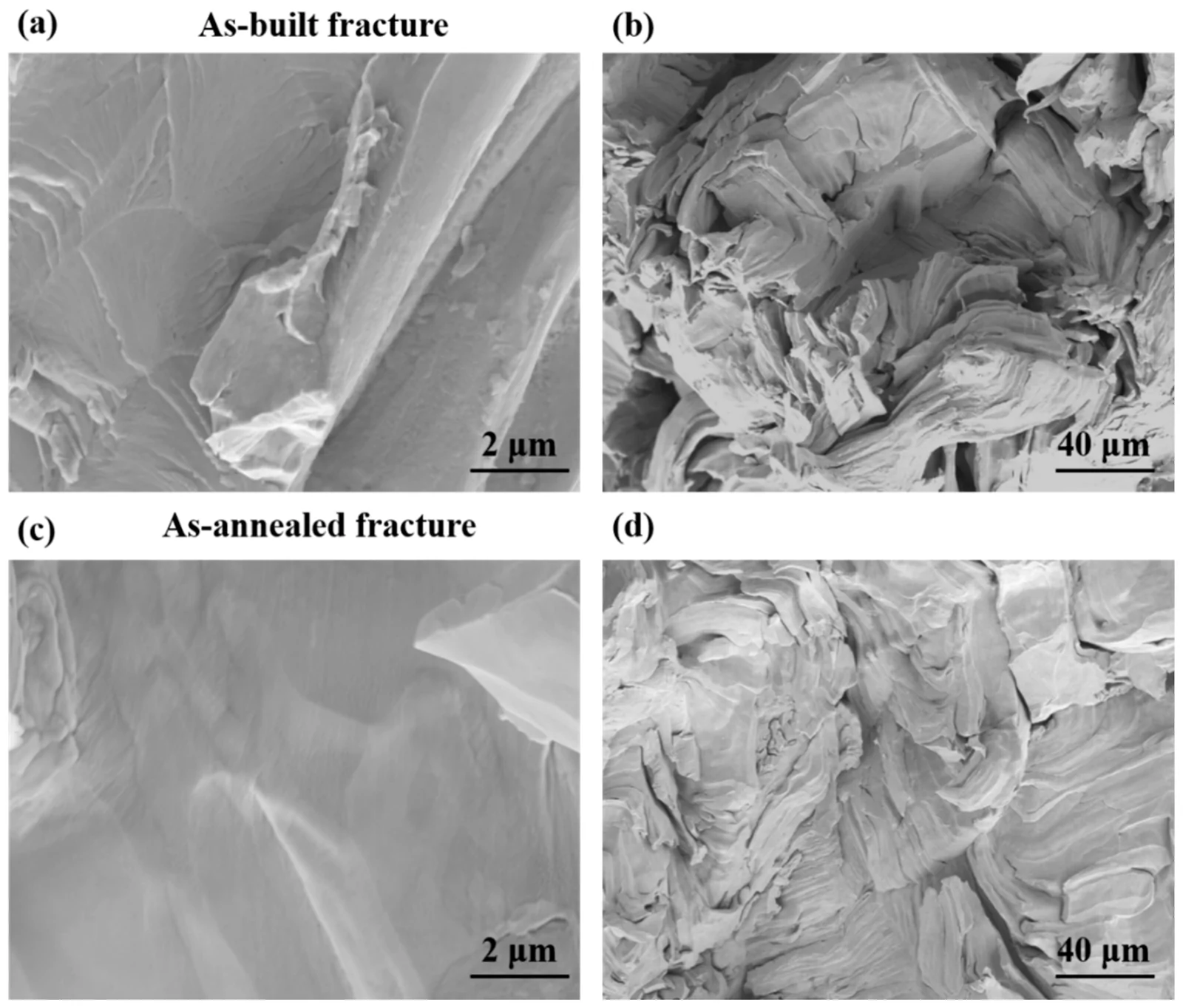
Compressive fracture surfaces of the (**a**,**b**) as-built and (**c**,**d**) annealed W-Mo alloys.

Figure 8 displays the room-temperature compressive fracture surfaces of the as-built and annealed W-Mo alloys. As shown in Figure 8a,b, the as-built specimen exhibits some intergranular fracture after compression, indicating relatively low grain boundary strength, which is mainly attributed to the high brittleness induced by the high residual stress. In addition, the dominant fracture feature consists of tortuous but continuous cleavage steps, corresponding to the grain morphology. After annealing (Figure 8c,d), the fraction of intergranular fracture in the W-Mo alloy decreases significantly, primarily owing to the reduction in residual stress and changed texture level. Meanwhile, the density of cleavage steps increases markedly, suggesting an improvement in the plastic deformation capability of the grains.

## 4. Discussion

For the as-built W-Mo alloy, a high density of <111>-oriented grains are aligned parallel to the building direction, which differs from the typical <100>-dominated texture commonly observed in additively manufactured alloys. During the LPBF process, the deposited layers or the substrate will be remelted. This remelting eliminates the nucleation barrier for solidification, and subsequent solidification proceeds via epitaxial growth on the partially melted grains [37]. A planar solidification front leads to the formation of elongated columnar grains, which is very common in LPBF-fabricated materials [38]. Initially, grain growth is strongly influenced by the crystallographic orientation of the parent grains. However, because crystals have a preferred easy-growth direction aligned with the heat flow direction, a competitive growth process occurs among grains. The competitive growth mechanism becomes more significant when the interlayer rotation angle is changed, as is the case with the 67° layer rotation angle employed in this work. Altering the layer rotation angle changes the direction of heat flow, and a small number of remelted grains will possess the most favorable crystallographic orientation, making the competitive grain selection more pronounced and, consequently, leading to a stronger texture. Such alternating cooling patterns induced by the layer rotation readily promote the development of <111> fiber textures in alloys such as Ta and W [39].

After annealing, the fraction of the <111>//BD orientation increases owing to its higher amount of stored energy. For tungsten, the <111> orientation exhibits superior mechanical properties compared with the <100> orientation, and cracks more readily propagate and expand along the cleavage planes in the <100> orientation. This is also evident from the compression fracture surfaces, the annealed W-Mo alloy with a higher <111> fraction displays finer cleavage facets, whereas the as-built alloy with a higher <100> fraction exhibits larger cleavage facets, primarily because the <100> texture corresponds to the preferred cleavage plane of tungsten [40]. When cleavage facets are larger, the probability of crack deflection during the propagation of microcracks along cleavage planes is lower, and cracks can propagate more easily. This is consistent with the fracture morphology and leads to inferior compression performance. On the one hand, stress-relief annealing homogenizes the dislocation distribution, thereby reducing the crack initiation susceptibility induced by dislocation pile-ups. On the other hand, the presence of residual thermal stress makes the material more prone to cracking. When voids or microcracks are generated during loading due to deformation, the combination with high residual stress accelerates crack nucleation and propagation [41].

The yield strength $\sigma_{y}$ of the as-built W-Mo alloy can be estimated by the following formula:

(5) $$\sigma_{y}=\sigma_{0}+\sigma_{gb}+\sigma_{d}$$

where $\sigma_{0}$ is the Peierls-Nabarro stress (~640 MPa for W [39]).

Grain refinement strengthening in metallic materials is commonly described by the Hall-Petch relationship, where the yield stress scales inversely with the square root of grain size. The Hall-Petch formula is expressed as follows [39]:

(6) $$\sigma_{gb}=kd^{-\frac{1}{2}}$$

where *k* is the Hall-Petch coefficient and *d* is the mean grain diameter (μm). For bcc refractory metals, *k* is taken as 210, and the average grain size of the as built W-Mo alloy is 9.74 μm. Through approximate calculation, the grain boundary strengthening contribution was determined to be 67 MPa.

Dislocation strengthening is quantified by the Bailey-Hirsch equation, which describes the strengthening effect arising from elastic interactions among dislocations and reflects the relationship between strength and dislocation density. *σ_d_* can be calculated using the following formula [39]:

(7) $$\sigma_{d}=MaGb\rho^{\frac{1}{2}}$$

where *M* = 2.733 is the Taylor factor for bcc metals [42], a is a constant = 0.5 [43], *G* = 160 GPa is the shear modulus of tungsten [44], and *b* refers to the absolute Burgers vector and *b* = 0.274 nm for BCC W. Here, ρ represent the dislocation density in as-built W-Mo. For the as-built W-Mo alloy, the EBSD-measured dislocation density is ρ = 0.72 × 10^14^ m^−2^. The calculated value can be roughly estimated as 177 MPa.

Based on the above calculations, the theoretical yield strength of the as-built W-Mo alloy is 884 MPa, which is in reasonable agreement with the experimentally obtained result.

## 5. Conclusions

In this study, highly dense W-15Mo alloys were successfully fabricated and subsequently subjected to stress-relief annealing. The main conclusions are as follows:

(1) The W-15Mo alloy powder, the as-built W-15Mo alloy, and the annealed W-15Mo alloy all consist of a single BCC-W phase, indicating that Mo is completely dissolved in the W matrix without precipitation. Both the as-built and annealed W-15Mo alloys exhibit a highly dense microstructure.
(2) The as-built W-15Mo alloy shows good thermal stability, with only a slight increase in grain size after annealing, and the grains remain predominantly elongated columnar. The fraction of the <111>//BD texture increases after annealing, leading to a higher dislocation density.
(3) Stress-relief annealing enhances the overall mechanical properties of the as-built W-15Mo alloy, with the compressive yield strength, ultimate compressive strength, and compressive strain increasing to 879.5 MPa, 1257.3 MPa, and 16.6%, respectively. Fracture surface analysis reveals that the stress-relief treatment effectively strengthens the grain boundaries and improves the ductility of the material.

## Figures and Tables

**Table 1:** Robust process design using an L_16_ orthogonal array.

Run	Laser Power (W)	Scan Speed (mm/s)	Energy Density (J/mm^3^)	Relative Density (%)		
				Experiment	Predicated	% Error
1	200	200	333.33	96.83	96.92	−0.0887
2	200	400	166.667	95.45	95.50	−0.0487
3	200	600	111.11	93.32	93.42	−0.0988
**4**	**200**	**800**	**83.33**	**91.18**	**90.68**	**0.5012**
5	225	200	375.00	98.70	98.68	0.0173
6	225	400	187.50	98.91	97.94	0.9683
**7**	**225**	**600**	**125.00**	**95.52**	**96.54**	**−1.02**
8	225	800	93.75	93.72	94.48	−0.7598
9	250	200	416.67	98.64	99.13	−0.4942
**10**	**250**	**400**	**208.33**	**99.46**	**99.07**	**0.2978**
11	250	600	138.89	98.69	98.35	0.3398
12	250	800	104.17	97.62	96.97	0.6518
**13**	**275**	**200**	**458.33**	**98.41**	**98.27**	**0.1368**
14	275	400	229.17	98.96	98.89	0.0697
15	275	600	152.78	98.34	98.85	−0.5072
16	275	800	114.58	98.18	98.14	0.0358

**Table 2:** S/N ratio response table for relative density.

Level	Laser Power	Scan Speed
1	39.48	39.84
2	39.71	39.84
3	39.88	39.69
4	39.87	39.57
Delta	0.40	0.27
Rank	1	2

**Table 3:** Results acquired from ANOVA—relative density.

Source	DF	Seq.SS	Adj.SS	Adj. MS	F	P	Percentage of Contribution (%)
Laser power	3	50.460	50.460	16.820	10.63	0.003	56.13
Scan speed	3	25.198	25.198	8.399	8.399	0.022	28.03
Error	9	14.238	14.238	1.582	-	-	15.84
Total	15	89.896		-	-	-	100

**Table 4:** The regression equation coefficients for relative density.

Coefficient	The Corresponding Value
a_0_	98.22
a_1_	2.21
a_2_	−1.59
a_3_	1.53

## Data Availability

The original contributions presented in this study are included in the article. Further inquiries can be directed to the corresponding authors.
